# Successful Treatment of Chronic Dysentery by Medication

**Published:** 1872-12

**Authors:** Richard B. Maury

**Affiliations:** Memphis, Tennessee; Professor of Theory and Practice of Medicine, Medical College of Memphis


### SUCCESSFUL TREATMENT OF CHRONIC DYSENTERY BY TOPICAL MEDICATION.

BY RICHARD B. MAURY, M D., Memphis, Tennessee.
*Professor of Theory and Practice of Medicine, Medical College of Memphis.*

Wherever dysentery prevails, the treatment of its chronic form has been found unsatisfactory to the last degree, and its prognosis exceedingly unfavorable.

Niemeyer declares that the subjects of this disease "usually die of marasmus and dropsy;" and according to Aitken "it is a malady, which, once fairly engrafted upon the system, never leaves it till life itself becomes extinct."

This too has been the experience, I am confident, of the physicians of the Mississippi valley.

Four years ago I adopted a plan of treatment in this disease which has been so successful in my hands that I wish to bring it prominently before the profession.

Case 1. In September, 1868, a young Jew, twenty-seven years of age, bed-ridden from chronic dysentery, came under my care. He had had constant medical attendance for three months, but was losing ground steadily. He was having daily five or six dysenteric stools, with much blood; was greatly emaciated, and very anemic.

The usual plan of treatment was tried for ten days, with no better success than had been obtained by those who had preceded me in the management of the case.

The patient was then placed in the left lateral position, used for making uterine examinations, and Sim's speculum, small size, was introduced into the rectum. Upon its anterior wall there was seen a round, superficial ulcer, half an inch in diameter. Upon the posterior aspect of the rectum there was found a much larger ulcer, whose lower margin was just an inch and a half from the anus. This ulcer involved the entire thickness of the rectal wall and the tissues intervening between the gut and the sacrum, so that a curved instrument was necessary to explore its cavity. A piece of sponge the size of a Guinea's egg could be buried out of sight in this cavity. No other ulcerations were visible, and the mucous membrane, remote from these lesions, was nearly normal.

After carefully cleansing the surfaces with cotton wool, a solution of nitrate of silver, ʒ ii to ounce of water, was applied to the ulcer and to the walls of the cavity. As the patient suffered much pain from this treatment, a hypodermic injection of morphia was given, and he was again put to bed. Immediate improvement followed. The movements from the bowels became less frequent and more natural, and the blood rapidly disappeared. In four days the treatment was repeated, and was followed by steady improvement. Five applications in all, were made to the diseased surfaces. The ulcer healed, and the cavity was rapidly filled with granulations, so that in five weeks the rectum presented a perfectly healthy surface, and th patient was soon able to resume business. The cure was perfect and permanent.

I was apprehensive that he would have a stricture of the rectum, but two years afterwards he was still in excellent health. Since then I have lost sight of him.

CASE 2. On the 20th March, 1870, a gentleman who had been planting in the swamps of Arkans s, fell into my hands for the relief of a chronic dysentery of thirteen months' standing. He was almost literally a walking skeleton. I administered a dose of oil, and next day examined the rectum. Found a single ulcer on its lateral and anterior wall about two inches from the sphincter. The mucous membrane was inflamed as far as the eye could reach. Five applications of the solution of nitrate of silver were made at intervals of four days. Improvement was marked from the beginning. The blood disappeared entirely from the stools after the third application, and did not return. In thirty days he was well. Heard from a year afterward; had had no return of the disease.

CASE 3. I treated Mr. H. E., æt. thirty-five years, for acute dysentery, from 28th June to 3d August, 1870. During my absence from home another physician was called to his case, and treated him until the 25th November following, when I was again sent for.

I found, upon rectal examination, six small ulcers of round or oval outline, upon the anterior and posterior walls, the mucous membrane for some distance around them being swollen and hyperæmic.

Four applications of nitrate of silver were made at intervals of a week. Marked improvement followed each one, and the patient was dismissed cured early in January, 1871. He has been under observation to the present time, and is in robust health. In this case, as in that which follows, diarrheal discharges took the place of the dysentery, and were troublesome for a little while after the ulcers were healed. They required no further treatment, however, than rest and a carefully regulated diet.

CASE 4. Mr. O. M. P., after suffering seven months from dysentery, came to me November 28th, 1870. Had been under the care of a skillful physician the greater portion of that time.

Was much emaciated and entirely incapacitated for business. Had half a dozen mucous and bloody stools a day, with frequent abdominal pains. A rectal examination was at once made, and a condition of things discovered almost identical with that found in case three. Four applications of nitrate of silver were made at intervals of a week, the patient not being confined to the house, and walking to my office for treatment.

By January 1st, 1871, the ulcers were healed, the rectum of normal appearance, the general health greatly improved, and the patient in fine spirits. The bowels were, however, a little disposed to diarrhœa, and so continued for nearly two months, but improvement was steady, and complete recovery eventually attained.

Case 5. Mrs. G., æt. thirty-five years, had suffered for two months with dysentery when she consulted me in May, 1871.

Internal medication at my hands having failed to relieve her, as it had done under the care of other physicians, a rectal examination was made June 1, 1871. No ulceration was found, but as far as the eye could reach the entire mucous membrane was found to be hyperæmic, swollen, covered with a thin mucinous material, and exhaling a bloody serum which ran out of the bowel through the speculum. Three applications of nitrate of silver, at intervals of four days, sufficed for the cure of this case. Several months afterwards she fell under my care again with hepatic abscess, from which she eventually died. The abscess was opened externally, and also discharged its contents into the bowel.

Case 5. Mr. S., of Memphis, thirty-five years of age, a sufferer from chronic dysentery of two years' standing, came under treatment June 20, 1871. He was so feeble and emaciated that he could not stand, and had been confined to bed for several months. The rectum was pale and bloodless in appearance, and its mucous membrane seemed to be entirely destroyed. One application of nitrate of silver was made, but without benefit. He died four days afterwards.

Case 7. H. A. G., æt. thirty-seven, came under treatment 17th September, 1871. He had been suffering from dysentery since the preceding April, and had been under treatment for the same in this city and in Chicago. His occupation was that of superintendent of a cotton shed—an occupation which necessitates great fatigue and exposure, because he has to stand for many hours daily on cold, damp ground. He could not possibly give up his occupation, as advised, and therefore unusual difficulties were encountered in the treatment. Upon examination, the surface of the rectum was found to be covered here and there with patches of "punched-out-looking ulcers with bloodless bases, and thin, anemic edges."—(Aitken.) From these patches a bloody serum constantly oozed.

His condition was critical at the time of his coming under treatment. Most men, under the same circumstances, would have abandoned business and taken to bed.

Eleven cauterizations with the solution of nitrate of silver ʒ ii to the ounce were made at intervals of four or five days, and after many reverses the patient was discharged completely cured on the 10th December, 1871.

He has continued to be in good health up to the present time, December 1, 1872.

Special difficulties, which delayed the cure, were encountered in this case, from the character of the patient, who was very headstrong and intractable. Severe tenesmus sometimes followed the treatment in this case, for the treatment of which a suppository of opium was used. Very often a half grain of morphia was administered just before the treatment.

Case 8. During the months of July and August, 1872, I treated my eighth case, that of a lady thirty-seven years of age, suffering from dysentery of seven months standing. As this case presented no details of unusual interest, I shall not report it at length. During the treatment the patient suffered twice from the invasion of acute dysentery with high fever, which very nearly carried her off. She is now in excellent health.

In addition to these cases, my partner, Dr. R. W. Mitchell, has treated quite a number upon the same plan, and with the same good results.

There is no other treatment for chronic dysentery that I am acquainted with, from which any such results as those here reported, could have been obtained.

In all these cases a great variety of treatment had been tried before they came under my care. While undergoing this treatment they received no other.

A theoretical objection will be raised at once against this method, that the disease is by no means limited to the rectum in a majority of the cases of chronic dysentery, and that it is impossible to make topical application of remedies to any other portion of the bowel.

Theoretical objections are worth nothing if the practical results are satisfactory. But in the present instance, I believe a rational explanation of the good effect of local applications, is to be found in the physiology of the act of defecation.

In health, the accumulation of fæces in the rectum makes an impression upon the nerve centres through the afferent nerve filaments. The result of this impression is the transmission of motor impulses through the efferent nerves to the muscular fibres of the entire intestinal tract, and especially to those of the rectum.

The action of these fibres together with the coöperative agency of the adominal muscles, constitutes the act of defecation.

In dysentery fæcal distension is not the cause of the intestinal movements. In consequence of inflammation, innervation becomes so deranged that a few spoonfuls of bloody mucus in the rectum provoke muscular contractions; and the tenesmus and frequency of the dysenteric discharges will depend upon the proximity of the inflammation to the sphincter. Irritation of the nerve filaments in this locality stimulates reflex movements, which disturb the whole intestinal tract. If you can quiet the irritation in the rectum, or if you can destroy the irritability of the mucous membrane for a time, you not only give rest to the rectum but to the entire colon also.

A striking illustration of the truth of these views is furnished by the following case:

Mrs. B., under my care in 1869, had pelvic abscess, the result of pelvic cellulitis, following her confinement. This abscess opened into the rectum.

After the purulent discharge had continued for ten days, the irritation produced in the rectum, by the opening of the abscess, seemed to be communicated to the entire intestinal tract, and an uncontrollable diarrhœa set in, which threatened to carry her off at once. The discharges then became small and dysenteric, and the call to stool was incessant.

Opiates by the stomach, and by the rectum, in the form of injections and suppositories, having been administered in large doses without effect, I insisted upon a rectal examination and succeeded in bringing into view a ragged opening with sloughing, ash-gray colored edges on the posterior and lateral aspect of the rectum, about two inches above the anus. This opening was about an inch in diameter and the discharge was of a grayish, yellow color, and of an indescribably offensive odor.

Through the speculum, I passed, on the end of a curved sponge holder, a sponge saturated with a strong solution of nitrate of silver, into this opening, and wiped it out well several times, as far as it was possible to carry the instrument.

Very great and almost immediate relief followed this operation. Two days afterward it was repeated, and with the same good result. Intestinal irritation was at once quieted by this treatment, and, with the aid of a half dozen opium suppositories, and the daily injection of a weak solution of carbolic acid in water, the patient improved rapidly and soon afterward returned to her home in Mississippi.

It will be remembered then, that the walls of the rectum constitute a surface upon which must be made the sensory impressions which are to originate motor impulses for the act of defecation. The effect of the application of nitrate of silver to this surface, when inflamed, is to allay irritability—to lessen its susceptibility to impressions. The bowels thereby are quieted, digestion is undisturbed, and the ulcers are enabled to take on a healthy and reparative action.

If we go beyond the bowels, we shall find analagous facts in the diseases of other organs. In cases of chronic ulcer, the stomach is more apt to eject its contents by vomiting, the nearer the ulcer is to the cardiac sphincter.

Inflammation about the sphincter vesicæ, of pressure or a calculus there, produces violent muscular contractions of the whole organ.

The same principle is illustrated in the effects observed from an inflamed and elongated uvula. Here an irritation at the outlet of the respiratory tract disturbs the entire tract; the removal of the irritant sets it at rest.

In conclusion, then, I will add, that I believe that the treatment here described is applicable to all cases of dysentery which have become chronic; and according to my observation this will usually be after the lapse of about six weeks from the commencement of the attack.

I would recommend that a full dose of opium be administered always just before the caustic application is made to the rectum. We thereby lessen the pain of the treatment, and secure rest to the bowel for several hours.

